# Recent Advances in Enzymes for the Bioremediation of Pollutants

**DOI:** 10.1155/2021/5599204

**Published:** 2021-06-22

**Authors:** Seyyed Mojtaba Mousavi, Seyyed Alireza Hashemi, Seyed Mohammad Iman Moezzi, Navid Ravan, Ahmad Gholami, Chin Wei Lai, Wei-Hung Chiang, Navid Omidifar, Khadije Yousefi, Gity Behbudi

**Affiliations:** ^1^Department of Chemical Engineering, National Taiwan University of Science and Technology, Taipei City, Taiwan; ^2^Health Policy Research Center, Health Institute, Shiraz University of Medical Sciences, Shiraz, Iran; ^3^Nanomaterials and Polymer Nanocomposites Laboratory, School of Engineering, University of British Columbia, Kelowna, BC V1V 1V7, Canada; ^4^Student Research Committee, Shiraz University of Medical Sciences, Shiraz, Iran; ^5^Pharmaceutical Strategic Studies Center, Shiraz University of Medical Sciences, Shiraz, Iran; ^6^Biotechnology Research Center, Shiraz University of Medical Sciences, Shiraz, Iran; ^7^Department of Nanotechnology & Catalysis Research Center, University of Malaya, Kuala Lumpur, Malaysia; ^8^Department of Pathology, School of Medicine, Shiraz University of Medical Sciences, Shiraz, Iran; ^9^Department of Chemical Engineering, University of Mohaghegh Ardabili, P.O. Box 179, Ardabil, Iran

## Abstract

Nowadays, pollution of the environment is a huge problem for humans and other organisms' health. Conventional methods of pollutant removal like membrane filtration or ion exchange are not efficient enough to lower the number of pollutants to standard levels. Biological methods, because of their higher efficiency and biocompatibility, are preferred for the remediation of pollutants. These cost-effective and environment-friendly methods of reducing pollutants are called bioremediation. In bioremediation methods, enzymes play the most crucial role. Enzymes can remedy different types of organic and inorganic pollutants, including PAHs, azo dyes, polymers, organocyanides, lead, chromium, and mercury. Different enzymes isolated from various species have been used for the bioremediation of pollutants. Discovering new enzymes and new subtypes with specific physicochemical characteristics would be a promising way to find more efficient and cost-effective tools for the remediation of pollutants.

## 1. Introduction

The widespread use of chemicals in industries and militaries, inadequate waste disposal, and accidental leakage cause contamination of soil, water, and air. For instance, there are 34,000 contaminated sites just in Europe that need to be treated. These pollutants are hazardous for humans, other living beings, and even the biogeochemical cycle. Pollutants' stability, low solubility, and resistance to various physical, chemical, and biological degradation pathways are the main reasons for their toxicity [1].

Different physical and chemical methods for cleaning up pollutants have been used, such as oxidizing agents, electrochemical treatments, adsorption of pollutants, ion exchange, and membrane filtration [2]. Despite the adequacy of traditional methods for the high concentration of pollutants, they were not enough for lowering the amount of contamination to regulatory limits [3]. Various disadvantages of traditional methods for cleaning up pollutants include high cost, nonspecificity, and probable secondary contamination production; therefore, ecofriendly and biological methods, called bioremediation, gained interest [4].

Bioremediation is defined as processes and products that are cost-effective and practical to minimize pollutants in the source and diminish danger to the environment and human health [5]. Its main ways of degrading and detoxifying pollutants are through intracellular accumulation or enzymatic transformation [4]. Pollutant properties (i.e., chemical structure, hydrophobicity, and polarity), environmental conditions (i.e., temperature, pH, and redox condition), and soil features (i.e., aggregation, thickness, dissolved organic matter, and pollutants aging) affect biological degradation and contaminants availability [6].

Enzymes are the most efficient bioremediation tools and progress all chemical changes on pollutants. Enzymes' specificity is usually broad enough to act on different molecules with similar structures. Moreover, it is possible to engineer the enzymes for enhancing their stability and efficiency for special conditions or particular substrates [7, 8]. Omics technologies have a significant role in these developments [2].

Using enzymes in bioremediation could be either individually that the isolated enzyme used and added to the contaminated area or as a whole cell, e.g., bacteria, fungi, or algae. In a second way, continuous aeration, inoculation, and nutrition are necessary. Besides, environmental conditions should be prepared for microorganisms living, even though there might still be some toxic compounds in the environment that inhibit microorganisms' activity [1, 9]. The use of individual enzymes has some advantages in comparison with microbial whole cell including greater specificity, more straightforward handling and storage, standardizable activity, more mobility as a result of smaller size, being active in the presence of high concentrations of toxic compounds, and biodegradability that inhibits persistence and recalcitrance [1, 10, 11]. This approach is much more efficient for extracellular enzymes and cofactor-independent enzymes [12, 13].

Enzyme production in the natural environment is low, while it is possible to increase the produced enzyme under controlled conditions. On the other hand, recombinant DNA technology and gene engineering provide many opportunities to produce more efficient and more enzymes [14]. Moreover, nanotechnology offers some tools to increase enzymes' stability by decreasing sensitivity to mechanical stress, preserving the third structure of enzymes, and protecting them against proteases [9].

**Figure 1:**
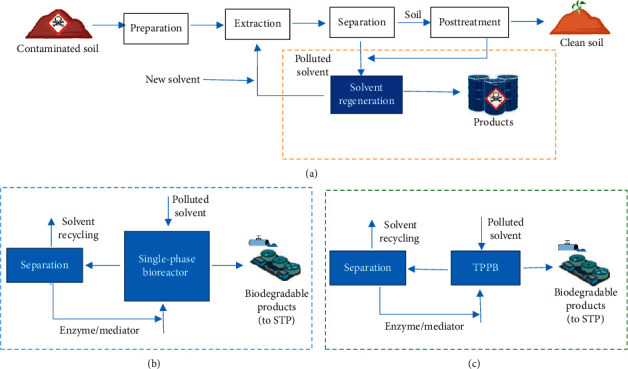
A representative scheme of different methods of soil remediation. (a) Conventional *in situ* remediation. (b) Using a single-phase bioreactor for solvent extraction. (c) Using a two-phase bioreactor for solvent extraction. Adapted from [1].

Enzymatic bioremediation could be *in situ* or *ex situ*. In *in situ* methods with the least disturbance in the environment, the free or immobilized enzyme (adsorbed enzymes on mineral supports that minimize the loss of enzymatic activity) is added to the soil. This approach is less expensive because of no need for excavation and transportation of soil. *Ex situ* methods are feasible for highly contaminated soils with toxic pollutants or when fast action is essential. During this procedure, soil was excavated and treated in different bioreactors in the best condition for enzymes' activity [1] (Figure 1).

Different enzymes like mono- or dioxygenases, halogenases, peroxidases, phosphotriesterases, hydrolases, transferases, and oxidoreductases from various species of bacteria, fungi, algae, and plants have been used for the bioremediation of pollutants [10, 15]. We try to review the most essential enzymes for the bioremediation of pollutants and insight into their mechanism of action.

## 2. Enzymes for Organic Substrates

Large amounts of organic pollutants, including herbicides, pesticides, dyes, drugs, and plastics, pollute the air, soil, and water every year. Polymers, aromatic molecules, polycyclic aromatic hydrocarbons (PAHs), chlorinated hydrocarbons, steroids, and organocyanides are the most organic compounds that need to be cleaned up worldwide. Their stable structure is the main reason for their toxicity.

### 2.1. Hydrolases (EC 3)

Esterases, nitrilases, aminohydrolases, lipase, cutinase, and organophosphorus hydrolase are among the hydrolase enzymes used in the bioremediation of different chemicals such as herbicides, pesticides, organophosphorus compounds, nitrile compounds, and polymers [1, 2]. We would review some of them shortly as follows.

### 2.2. Esterases (3.1)

Esterases catalyze the cleavage of ester bonds in different chemicals like organophosphorus herbicides and pesticides, diethyl glycol adipate, polyurethanes, and aromatic and aliphatic polyesters. *Escherichia coli* and *Pichia pastoris* are two bacteria that express and colonize the thermostable kind of enzymes. Moreover, a subgroup of esterases found in *E. coli* is active in a cold environment and can act on phthalate esters [2].

It is worth noting that the product of esterase reaction with organophosphorus compounds, 3,5,6-trichloro-2-pyridinol (TCP), is metabolized later to less toxic chemicals by aminohydrolase (EC 3.5) [2].

### 2.3. Nitrilases (EC 3.5.5.1)

Triple bonds between carbon and nitrogen (nitrile group) of herbicides, polymers, and plastics are hydrolyzed stereo-, regio-, or chemoselectively by nitrilases to carboxylic acid and ammonia. Many species can express these enzymes, including *Streptomyces* sp., *Fusarium solani, Rhodococcus rhodochrous, Aspergillus niger, Bacillus pallidus,* and *Pseudomonas fluorescens*. Moreover, an evolution approach on *Alcaligenes faecalis* tends to isolate a nitrilase that was active in the broader range of pH. Besides, *P. fluorescens* nitrilase's gene expressed in *E. coli* is probably the most hopeful nitrilase [16, 17]. Cyanide dihydratase (EC 3.5.5) is one of the nitrilases and degrade cyanide into formate and ammonia. *Pseudomonas stutzeri* and *Bacillus pumilus* are two species that express this enzyme. Furthermore, fungal cyanide hydratase (EC 4.2.1.66), isolated from *Fusarium lateritium, Neurospora crassa*, and *Gloeocercospora sorghi*, and some other species, is another cyanide-degrading enzyme that metabolizes it to formamide [18]. These enzymes are promising for the bioremediation of wastewaters from coal coking and metal-plating baths [17].

### 2.4. Organophosphorus Hydrolase (EC 3.1.8.2)

Organophosphate compounds were developed and used as pesticides and in warfare and even as a drug since 1937. They are neurotoxic, and after a while, they were more than that soil microbiota could remedy all of them. Organophosphorus hydrolase (also known as phosphotriesterase) is one of the enzymes that can serve for organophosphorus compounds bioremediation. It is mostly isolated from *Pseudomonas diminuta*, although its fungal form is expressed in *Aspergillus niger* and *Penicillium lilacinum*. It can act on P-S, P-O, and P-F bonds. This enzyme has Zn^2+^ as a cofactor in its native form, while assays showed that substitution of Co^2+^ provides the most potent activity against paraoxon [19]. This enzyme has the fastest catalytic rate and is the most promising enzyme for engineering activity against organophosphates [20].

#### 2.4.1. Peroxidases

*(1) Ligninolytic Peroxidases*. Ligninolytic enzymes are a family of enzymes with broad applications in bioremediation. This group of enzymes produced by white-rot fungi (WRF) is in the condition of nutrient limitation known as “ligninolytic.” Also, lignocellulosic materials can be an inducer for the production of these enzymes [21]. Due to the high nonspecificity and high nonstereoselectivity of these enzymes, they can degrade a wide range of recalcitrant compounds [22]. They degrade chemicals by pseudo-first-order kinetic via a free-radical-based chain reaction using H_2_O_2_ and molecular oxygen [21–24].

Ligninolytic enzymes can be categorized into four main enzymes, including laccase (LAC), lignin peroxidase (LiP), manganese peroxidase (MnP), and versatile peroxidase (VP).

**Figure 2:**
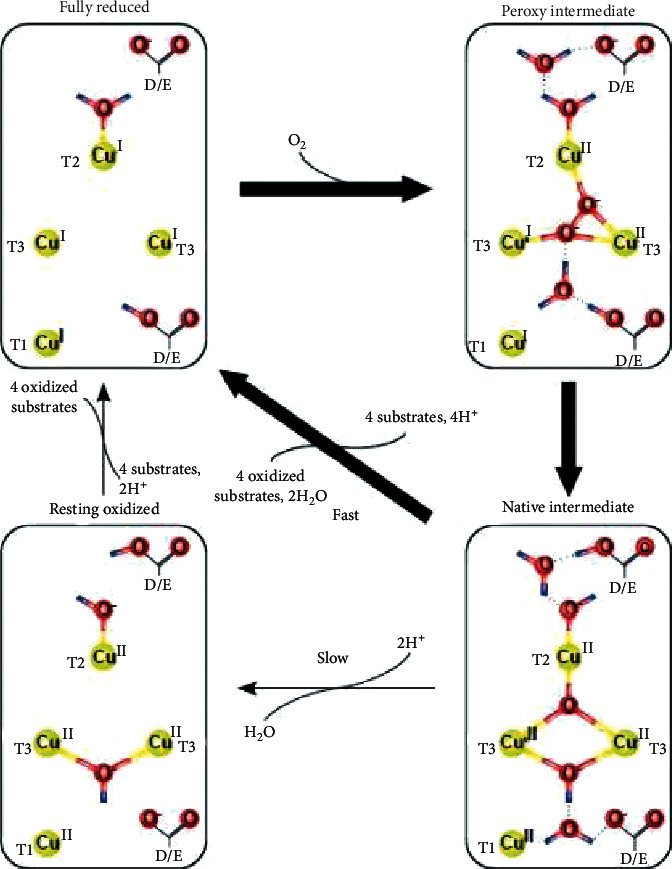
General reaction mechanism of bacterial laccases. Adapted from [26].

*(2) Laccase*. For oxidizing phenolic compounds, PAHs, dyes, and pesticides benzenediol: oxygen oxidoreductase, known as laccase, is a suitable enzyme. As an oxidase, laccase substrates go through one of the following pathways: (1) cleavage of aromatic rings, (2) polymerization, and (3) degradation of covalent bonds between monomers. Four atoms of copper are the principal part of the reaction, and oxygen is the last electron receptor [2, 25]. The mechanism of the reaction is shown in Figure 2.

Laccase is first discovered in different fungi species like *Panus conchatus* and *Polyporus* sp. Later on, laccase was found in *Azospirillum lipoferum,* as the first bacteria species. Laccase is produced in different Gram-positive bacteria, including *Bacillus*, *Geobacillus, Aquisalibacillus, Lysinibacillus, Staphylococcus*, and *Streptomyces*. Many bacteria produce laccase extracellularly, while some others are unable to secrete the enzyme. Bacterial laccase is more resistant to extreme temperature and pH conditions [1, 25].

There are two kinds of laccase, white and blue. The main difference between these is that blue laccase is dependent on a “mediator” for the degradation of nonphenolic substrates. “Mediator” is an intermediator that laccase oxidizes and turns into oxidized radicals that react with high redox potential or bulky substrates. ABTS (2,20-azino-bis (3-ethylbenzothiazoline-6-sulfonic acid)) and N-heterocycles with N-OH such as violuric acid, N-hydroxybenzotriazole, and N-hydroxy-N-phenylacetamide have been used as effective mediators [25].

Every year, approximately 7 × 10^4^ − 1 × 10^7^ tons of dyes penetrate the environment [25]. Laccase is used for dyes remediation. As an example, a *Bacillus licheniformis* LS40-derived laccase can decolorize azo, indigo, and anthraquinone dyes by 80% within one hour in the presence of acetosyringone as a mediator [27].

PAHs are xenobiotic pollutants because of their low solubility and degradation rate. Laccase can convert PAHs to their less toxic quinine form and CO_2_. There are some examples in Table 1. Notably, laccase can degrade some drugs such as diclofenac and mefenamic acid in acidic pH [25].

*(3) Lignin Peroxidase*. Lignin peroxidases (LiPs) are a group of heme-containing monomeric enzymes. Their weight ranges between 38 and 43 kDa [30, 31] with iron in the ferric state [21, 32]. LiPs with their high redox potential [32] are capable of breaking alpha and beta carbon bonds, catalyzing the degradation of phenolic and nonphenolic compounds, demethylation, and opening aromatic ring of dyes [33]. LiPs have a high redox potential for oxidizing nonphenolic structures [31].

**Figure 3:**
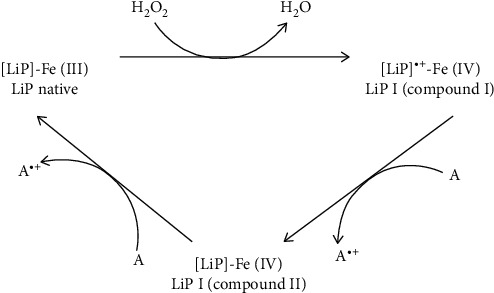
Lignin peroxides catalytic reaction. Adapted from [35].

LiP activity increases in the presence of H_2_O_2_ as an electron acceptor. However, high concentrations of H_2_O_2_ could damage the LiPs [32]. In the first step of the reaction, Fe^3+^ binds to H_2_O_2_ and oxo-ferryl intermediate named compound I forms. Then, compound I, by a donation of one electron from the substrate, reduces to compound II, finally; by another electron donation from the substrate, iron in heme returns to its ferric resting state, and the enzyme renews to its initial form [31, 34]. In this three-step reaction, the reduction of compound II is the rate-limiting step (Figure 3) [36]. Due to this slow reduction rate, compound I is available for reaction with H_2_O_2_ and the formation of a complex between LiP and superoxide (compound III) inactive enzyme [36].

Veratryl alcohol is a secondary metabolite that can play essential roles in this oxidizing reaction. Veratryl alcohol can be the mediator in the electron transfer reaction; it can play a role in the catalytic cycle of LiP by an oxidizing terminal substrate. Vertaryl alcohol can also prevent the formation of compound III and, if compound III is established, reduce it to its native form [36].

Many WRFs produce LiPs such as *Phanerochaete chrysosporium*, *Trametes versicolor*, *Bjerkandera adusta*, *Phlebia radiate*, and *Ganoderma lucidum* [22, 31].

Many technologies have been applied to enhance activity and increase catalytic characteristics of LiPs, such as LiP entrapment in calcium beads [37].

**Figure 4:**
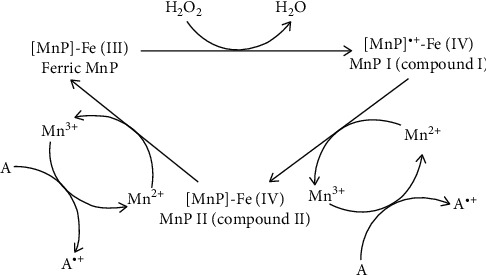
Manganese peroxidase catalytic reaction. Adapted from [35].

*(4) Manganese Peroxidase*. Manganese peroxidases (MnPs) are heme-containing glycol proteins with weight ranging from 32 to 62.5 kDa [38]. Like other ligninolytic peroxidases, MnP uses H_2_O_2_. By using H_2_O_2_, MnP can oxidase Mn^2+^ to Mn^3+^. The first step of the reaction is binding an oxygen atom of H_2_O_2_ to Fe^3+^ of heme. Then, by two-electron transfer from Fe^3+^ to peroxide Fe^4+^ oxo-porphyrin, compound I radical forms. Then, compound I binds to monochelated Mn^2+^ and Mn^3+^ and compound II forms. Finally, by oxidizing another Mn^2+^ to Mn^3+^, compound II reduces and the enzyme with Fe^3+^ reforms (Figure 4) [32, 36].

Aliphatic organic acids such as lactate and oxalate can induce Mn^2+^ oxidation rate, and Mn^3+^-acid chelates have a higher redox potential. MnP activity increases in the presence of glutathione and unsaturated fatty acids, such as tween 80. Many techniques have been utilized to immobilize and enhance the efficacy of bioremediation with MnP, such as making calcium alginate beads and carbon nanotubes [39–41].

MnP can remediate PAHs and nitroaromatic compounds [36, 42], azo dyes [43], and endocrine-disrupting chemicals such as bisphenol A and alkylphenols [44, 45]; moreover, with the contribution of mediators such as lipid and thiyl radicals, MnP is capable of oxidizing nonphenolic structures [34].

Many species of fungi are able to produce MnP, such as *Phanerochaete chrysosporium*, *Trametes versicolor*, *Irpex lacteus*, *Dichomitus squalens*, and *Ganoderma lucidum* [45, 46].

*(5) Versatile Peroxidase*. Versatile peroxidase (VP) is a heme-containing ligninolytic enzyme considered as a hybrid between LiP and MnP. VP has two active sites; therefore, it can oxidize both Mn^2+^ and veratryl alcohol by a similar mechanism to MnP and LiP, respectively [32, 47].

VP can oxidize both low and high redox potential compounds, polycyclic aromatic hydrocarbons, azo dyes, high molecular weight aromatics, and both phenolic and nonphenolic compounds and environmental pollutants [32, 47, 48].

VP production is less common in WRFs than MnP and LiP, but it can be found in some species such as *Pleurotus* spp. and *Bjerkandera* spp. [32].

#### 2.4.2. Horseradish Peroxidase

**Figure 5:**
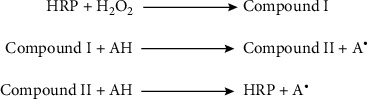
Horseradish peroxidase catalytic cycle. Adapted from [52].

Horseradish peroxidase (HRP) is an enzyme traditionally extracted and isolated from the root of horseradish (*Armoracia rusticana*). The most abundant isoenzyme found in the root of horseradish is C isoenzyme (HRPC). HPRC is 44 kDa heme-containing glycopeptide with 308 amino acids, an iron atom in the ferric state in protoporphyrin IX, and two calcium atoms in the central zone [49–51]. HRP catalyzes oxidative reaction using H_2_O_2_. In the presence of H_2_O_2_, the intermediate compound formed via two-electron oxidation. Then by an oxidable substrate, compound I reduces to compound II. Radical formation occurs via these reactions, and finally, the initial enzyme can be renewed by the reaction of compound II with another substrate molecule. In comparison with LiP compound, I and II are more electronegative in HRP (Figure 5) [52].

HRP is applicable for removing and remediating phenols, substrate phenols, and alkylphenols, aromatic amines [53, 54], azo dyes [55, 56], endocrine-disrupting compounds [54], and many other environmental pollutants.

Many techniques have been utilized to immobilize and enhance the efficacy of enzyme by nanotechnology [57–59]. Using horseradish root is a standard method; using fertile soil for horseradish cultivation to feed the population has raised concern in recent years [60]. To solve this problem and enhance the efficacy of the enzyme, many biotechnological methods have been experienced, such as recombinant production of HRP in *E. coli*, yeast, plants, and insect systems [61].

### 2.5. Cytochrome p450 Monooxygenase (EC 1.14.14.1)

**Figure 6:**
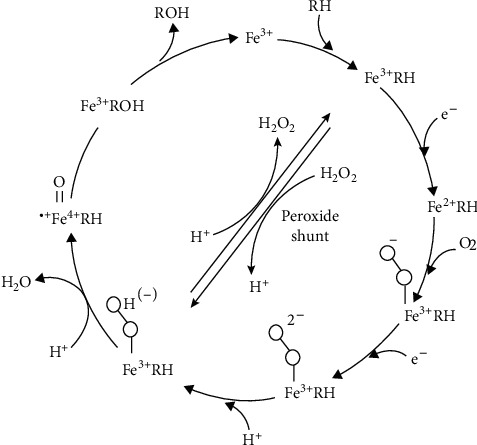
Cytochrome p450 reaction cycle. RH: substrate; ROH: product. Adapted from [63].

Cytochrome p450 monooxygenases (CYP) are a family of heme-containing enzymes that catalyze different reactions such as N-hydroxylation, N-dealkylation, O-dealkylation, oxidative dehalogenation, and hydroxylation of C-H bonds. CYP derives essential electrons for reactions from NADPH-cytochrome p450 reductase, and the latter enzyme derives electrons from atmospheric oxygen. So, the presence of a reducing agent like NAD (P) H or FAD is necessary [62]. The reaction cycle of CYP450 is shown in Figure 6.

CYPs are versatile enzymes expressed in various species of bacteria, fungi, plants, and animals. About 7000 different CYPs have been discovered till now. *Saccharomyces, Streptomyces, Basidiomycete, Dehalococcoides, Rhodococcus, Bacillus, Escherichia,* and *Salmonella* are among the genera that their CYPs are used for bioremediation [64, 65].

While bacterial CYPs are attractive because of their solubility, easy and low-cost production, and self-efficiency (their electron transfer reductases, e.g., FMN, FAD, and p450 monooxygenase, are on a single peptide), mammalian CYPs are membrane-bounded, dependent on a redox partner (e.g., NADPH) and have expansive applications [65]. Bacterial and eukaryotic CYPs can oxidize aliphatic hydrocarbons with 5–16 and 10–16 carbon lengths, respectively [66]. Notably, eukaryotic CYPs need modification at N-terminal, but prokaryotic ones are active in the native form [64].

Dioxins, PCBs (polychlorinated biphenyls), PCDDs (polychlorinated dibenzo-p-dioxins), PCDFs (polychlorinated dibenzofurans), PAHs, aliphatic hydrocarbons, and even Cr (VI) are pollutants that can be degraded and bioremedied by CYPs [1, 14, 65, 67]. In Table 2, the list of different CYPs and their substrates are shown. Immobilizing CYPs can improve their activity even to 10-folds higher than free enzyme. Besides, transgenic plants that can produce special CYPs are a way toward herbicide-resistant plants [65].

CYPs are interesting enzymes for bioremediation because of their wide range of substrates and diverse oxidative reactions. Among the limitations of using CYPs are their dependency on expensive cofactors, low stability, and low activity [68].

## 3. Enzymes for Inorganic Substrates

In the presence of toxic heavy metals, most of the microorganisms produce metal-binding peptides such as phytochelatins and metallothioneins, which reduce their toxicity via sequestration [70]. For example, phytochelatin synthase is the enzyme responsible for the production of phytochelatin that, in cooperation with GSH, accumulates heavy metals [71]. Among the limitations of these metal-binding proteins is their nonselectivity. To solve this problem, many microorganisms developed specific pathways for resistance against heavy metals [4]. Obviously, enzymes are the most critical part of these pathways; we would review some of these metal-specific enzymes as follows.

### 3.1. Arsenic

Arsenic is a heavy metal that exists in nature in organic and inorganic forms. The inorganic forms (As^3+^ (arsenite) and As^5+^ (arsenate)) are toxic and may cause enzyme inactivation, carcinoma, hemolysis, keratosis, gangrene, and neurological and cardiovascular diseases [72, 73]. Arsenate and arsenite convert to each other by arsenate reductase and arsenite oxidase through redox reactions. As^3+^ is more mobile and toxic. As^5+^ is the terminal electron acceptor in the absence of oxygen and reduces to As^3+^ [63]. Ferredoxin or glutathione would be the electron source [74]. This process enhances the solubility of As and eases leaching from soil [73]. The final As^3+^ is excreted through efflux pumps, ArsB and Acr3 [74]. Arsenite oxidase converts As^3+^ to less toxic As^5+^ to be used either for a supplemental energy source or as an electron donor for CO_2_ fixation [74]. The final arsenate is immobile and would be retained by sediments [73].

The methylated form of arsenic is volatile and would be lost from the soil [73]. Interestingly, in methanogenic bacteria, As methylation is coupled with methane biosynthesis and can detoxify soil through this mechanism. Coenzyme M is the biocatalyst of this detoxification process [63].

**Figure 7:**
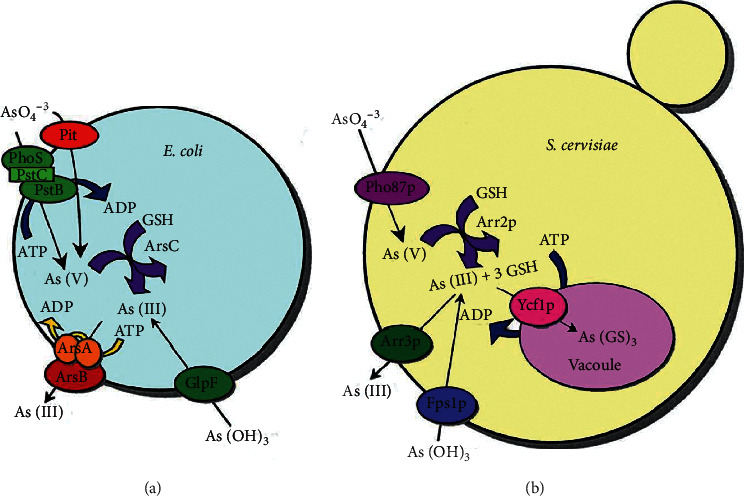
A strategy used for arsenic detoxification using *E. coli* and *S. cerevisiae* and their enzymes. Adapted from [63].

Many species can remedy As in different ways. The bacterial ones include *Acinetobacter* sp., *Pseudomonas* sp., and *Sporosarcina ginsengisoli* [75]. *E. coli, Bacillus idriensis*, and *Sphingomonas desiccabilis* are engineered species for As bioremediation [72]. Some fungi, including *Rhizobium* sp., *Rhizopus* sp.*, Trichoderma* sp., *Aspergillus flavus*, and *Penicillium canescens*, are As bioremediators too [63, 73]. Moreover, some yeasts like *Saccharomyces cerevisiae* can reduce arsenate by ArsC (Figure 7), a protein that has As reductase activity. Algae genera, like *Hydrodictyon, Oedogonium, Rhizoclonium,* and even a plant, *Pteris vittata* from Pteridaceae, have the potential to be used for bioremediation [75].

### 3.2. Lead

Lead was found in a small amount in nature before industrialization. However, now, through gasoline burning, different Pb salts originate in and contaminate water, soil, and air [72]. Lead toxicity may cause anemia and appetite loss and gastrointestinal, neurological, and reproductive disorders [73]. Organoleads, especially tetraethyl lead and tetramethyl lead used in gasoline, are toxic forms of lead. They are sensitive to photolysis and volatilization and degrade to dialkyl species. Though, some bacteria can degrade organoleads through bioremediation processes [76].

*Cupriavidus metallidurans* can remove Pb^2+^ ions with p-type ATPase and produce inorganic phosphate to sequester Pb^2+^ in the periplasm [76]. *Staphylococcus epidermidis* can biomineralize Pb^2+^ by carbonate. Urease enzymes form different carbonate crystalline Pb^2+^. It can be mineralized as oxalate and pyromorphite, too [77]. *Agaricus bisporus, Rhizopus nigricans, Penicillium canescens, Penicillium chrysogenum, Saccharomyces cerevisiae, Aspergillus niger,* and *Aspergillus terreus* are among biotransforming organisms [72, 73]. Moreover, it is reported that *Arthrobacter* and *Phaeolus schweinitzii* can degrade trimethyl lead cations [78].

### 3.3. Mercury

**Figure 8:**
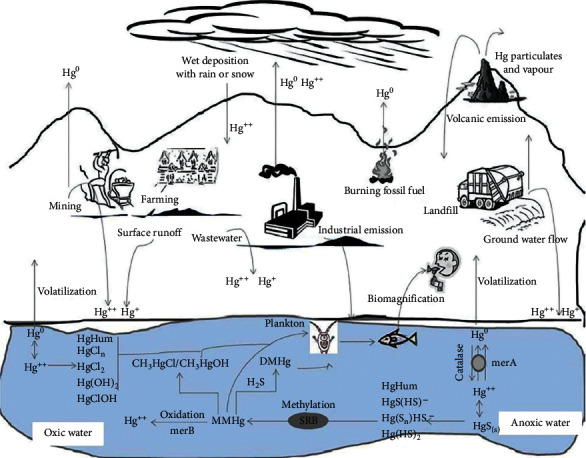
Mercury cycle in the environment. Adapted from [80].

Mercury is a heavy metal that is toxic in both organic and inorganic forms, although the organic form is more toxic. Hg toxicity would cause neurotoxicity, nephrotoxicity, allergies, and inability to speak [73, 79]. Hg is a rare element in Earth crust, but it spreads and pollutes soil and water because of different humic activities like gold mining, various measurement tools (barometer, thermometer, manometer, etc.), lamps, mercurial fungicides, paper manufacturing industry, and battery cells [72]. Its environmental cycle is shown in Figure 8.

Mercury exists in three forms: metallic mercury (Hg^0^), mercurous (Hg^+1^), and mercuric (Hg^2+^) forms. The most toxic form of Hg is mercuric chloride. Organic mercury can accumulate in living organisms and has an affinity for proteins' sulfhydryl groups. Inorganic mercury has the lowest toxicity because of its low solubility and high vapor pressure. Mercury-resistant bacteria (such as *Pseudomonas, Aeromonas, Staphylococcus, Escherichia, Citrobacter, Bacillus,* and *Rhodococcus*) can reduce toxic organic forms of Hg to less toxic metallic Hg. Mercuric reductase is the main enzyme that reduces Hg. The *mer* operon is the collection of mercury-resistance genes activated in the presence of an inducible concentration of Hg. Mercuric reductase in cooperation with FAD and NADPH, as electron sources, reduces Hg^2+^ to Hg^0^. The final metallic mercury is volatile and spreads to the atmosphere [80, 81]. Also, dimethylmercury is volatile and biomethylation can serve as a strategy for Hg bioremediation [73]. The *mer* operon-independent volatilization of mercury has been discovered, too, in *Shewanella oneidensis* [82].

Another enzyme that plays a role in mercury bioremediation is organomercurial lyase that breaks the carbon-mercury bonds in organo-Hg compounds [80, 81].

Various microorganisms such as *Rhizopus arrhizus, Penicillium canescens, Geobacter sulfurreducens, Pseudomonas putida, Acinetobacter calcoaceticus, Staphylococcus aureus,* and *Shigella flexneri* can remedy mercury [72, 80]. *Enterobacter, Pseudomonas*, and *Bacillus* are the most used genera for this purpose [83].

### 3.4. Chromium

**Figure 9:**
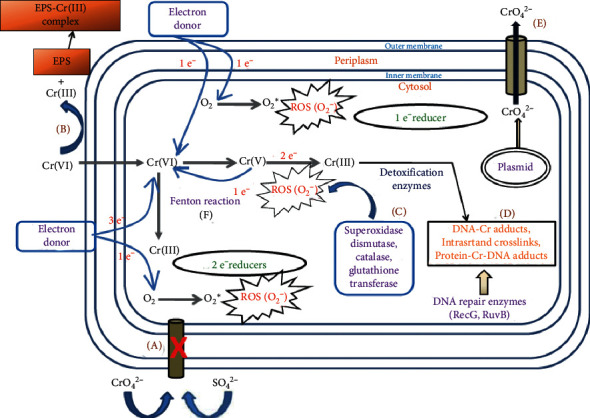
Chromate resistance mechanism in bacteria. (A) Mutation in sulfate uptake transporters. (B) Extracellular reduction of Cr^6+^ to Cr^3+^. (C) Intracellular reduction of Cr^6+^ to Cr^3+^ by chromate reductase. (D) Reducing oxidative stress and activation of repairing systems. (E) Outflowing of chromate from the cytoplasm. (F) Decreasing oxidative stress by activation of ROS scavenging enzyme. Adapted from [14].

Cr (VI) is the most toxic heavy metal because of its high oxidative potential causing cell damage and mutagenic, carcinogenic, and teratogenic effects [84]. The wide use of chromium and its compounds and mining exerts this pollutant to waters and soils. Bioremediation of hexavalent chromium is through reduction to trivalent species. *Pseudomonas, Bacillus, Escherichia, Shewanella, Enterobacter*, and *Thermus* are some genera that are resistant to Cr (VI) and can reduce it. The reduction of hexavalent chromium may occur through aerobic or anaerobic pathways [14]. In the anaerobic process, soluble cytoplasmic enzymes are involved and reduce hexavalent chromium in two steps. In the aerobic reduction of chromium, usually, Cr (VI) is a terminal electron acceptor, while in different species, NADPH, NADH, or formate serves as an electron donor. Chromate reductase, Ni-Fe dehydrogenase, and cytochrome c3 are among the enzymes reported to have hexavalent chromium-reducing activity [85]. Also, Fe^2+^ and S^2-^ produced in some bacteria can reduce Cr^6+^ even faster than chromate-reducing bacteria. The detailed mechanism of chromate resistance in bacteria is shown in Figure 9.

Nitroreductase, iron reductase, flavin reductases, and quinone reductases are bacterial enzymes that reduce Cr^6+^ [86, 87]. Mammals reduce this pollutant, too, by CYP, aldehyde oxidase, and DT-diaphorase. Some of these bacterial enzymes are extracellular, including nitrate reductases, flavin reductases, and ferrireductases [14].

## 4. Conclusion

In this review, we aim to provide an insight into the role of the enzyme in the bioremediation of pollutants. While many physical and chemical methods of treating contaminated soil and water are not efficient enough, bioremediation opens a new way to clean up toxic pollutants. Enzymes as practical tools of living organisms are an ecofriendly and bio-based strategy for bioremediation. Microorganisms exposed to contaminated sites and specific pollutants are fascinating sources for the isolation of active enzymes against those pollutants. Interestingly, we may find some enzymes in completely irrelevant places to pollutant sources. Discovering TCP-degrading enzymes and chlorpyrifos-degrading enzymes in the cow rumen microbiome is an instance for this claim [2].

Overall, using enzymes for pollutant bioremediation seems to be a cost-effective, efficient, and practical approach. Although there are still many ways to go, further studies and experiments on enzyme activity and mechanism of action and isolating new enzymes would be a promising way to reduce pollutants and make a healthier environment for humans and all other species.

## Figures and Tables

**Table 1:** Examples of laccase's pollutant bioremediation.

	Substrate	Bioremedied form	References
1	Acenaphthylene	1, 2-Acenapthalenedione	[28]
2	Acenaphthylene	1.8-Naphthalic acid	[28]
3	Bisphenol A	4-Ethyl-2-methoxy phenol	[29]
4	Benzo[a]pyrene	Methyl-3-hydroxy-8-methoxy-9, 10-dioxo-1-propylanthracene-2-carboxylate	[29]

**Table 2:** Cytochrome p450 subtypes and their substrate for bioremediation.

	Enzyme	Substrate	Reference
1	Rat CYP1A1	Dibenzo-p-dioxin (DD) and mono-, di-, and trichloro-DD	[65, 67]
2	F240A	2,3,7,3,8-Tetrachloro-DD	[65]
3	CYP101, CYP102, CYP1A1, CYP1A2, CYP1B1	PAHs	[65]
4	Dog CYP2B11	PCBs	[65]
5	CYP1A1, CYP1A2, CYP1B1	Low-chlorinated PCDDs	[68]
6	CYP5145A3	1-MCDD, 2-MCDD, 2,3-DCDD	[68]
7	CYP XplA	RDX (hexahydro-1,3,5-trinitro-1,3,5-triazine)	[69]

## Data Availability

The data used to support this study are available upon request.
